# Book Reviews

**Published:** 1904-07

**Authors:** 


A System of Practical Surgery. By Drs. E. von Bergmann, Berlin, P. von Bruns, Tübingen and J. von Mikulicz, Breslau. Translated and edited by William T. Bull, M.D., Professor of Surgery in the College of Physicians and Surgeons (Columbia University), New York, and Walton Martin, M.D., Instructor in Surgery, College of Physicians and Surgeon, New York. To be complete in five imperial octavo volumes. Volume I., pp. 936. With 361 engravings and 18 plates. Philadelphia and New York: Lea Brothers & Company. 1904. (Price, per volume: cloth, $6.00; leather, $7.00; half morocco, $8.50.)

The literature of surgery has been enriched during the past ten years by numerous contributions of value, consisting of text-books, encyclopedias, monographs and brochures, but it is doubtful if any one of them has excited more interest among surgeons throughout the world than the one under consideration. We need not go far to discover the reasons for this fact; it is an exposition of modern surgery from the German viewpoint; the authors are three most conspicuous surgeons representing the advanced thought of continental Europe; it is translated and edited by two American surgeons whose fame, especially that of Dr. Bull, is world wide. Moreover, while it is encyclopedic in character, it is exhaustive, or as nearly so as any general treatise can be.

The volume under consideration deals with the surgery of the head and contains 966 pages,—more than ever have been devoted to that subject in a single volume. It begins with injuries of the head before birth and during labor, and discusses all phases of injuries and diseases of the skull and its contents; then malformations, injuries, and diseases of the ear receive due attention; next malformations, injuries and diseases of the face, and plastic operations are considered; then follow in regular order neuralgias of the head; anomalies, injuries, and diseases of the salivary glands; injuries and diseases of the jaw; malformations, injuries and surgical diseases of the nose and its adjacent tissues; malformations, injuries and diseases of the mouth; and, finally, malformations, injuries and diseases of the pharynx.

The surgery of the brain, particularly for abscess, operative exposure of the mastoid cells, the surgical treatment of epilepsy, and plastic operations for cleft formations, are of particular interest, and all the newer methods of dealing with these several conditions are set forth with precision; indeed, this may be said of all the operative procedures described in the book. If, in addition, good printing and abundant high-class illustrations enhance the value of a book, assuredly this one is of the best.

---

Annual Report of the Surgeon-General of the Public Health and Marine Hospital Service of the United States. For the fiscal year 1902. Washington: Government Printing Office. 1903.

The material in this volume is of great interest to the sanitarian and public health officer. A portion of it also is of great interest to the clinician; for example, that part treating of the sanitorium for consumptives at Fort Stanton, New Mexico. The division of foreign and insular quarantine should be read by everybody who thinks this work is a sinecure, or that it is imperfectly performed. One of the items alone represents great labor—namely, the disinfection of 110,713 pieces of baggage at the Manila and Mariveles stations, 382 vessels also having been disinfected at the latter station. The Surgeon-General and his assistants are entitled to the greatest praise for the efficiency of their work, and for the faithfulness with which they guard all the avenues through which infectious diseases are liable to reach the United States.

The book is interestingly illustrated with half-tone plates showing various phases of the work, and may be said to be an object lesson in preventive medicine.

---

A Manual of General Pathology. For Students. By Sidney Martin, M.D., F.R.S., F.R.C.P., Professor of Pathology at University College; Physician to University College Hospital, London. Octavo, pp. 521. Illustrated. Philadelphia: P. Blakiston's Son & Company. 1904. (Price, $4.00.)

The arrangement made use of by this author is well adapted to the needs of the student, and is calculated to simplify the study of pathology as much as is consistent with a due regard to the importance of the subject. Beginning with inflammation, Martin takes the student through its various manifestations to infection, then to the degeneration and regeneration of cells and tissues; describes the changes in the circulation and respiration in disease; the changes in the blood occurring during disease processes; hemorrhage and pigmentation; the effects of disease of the liver, of the kidneys and the ductless glands; changes in metabolism; and, finally, the changes in the nervous system.

Martin is intensely interesting in his chapters relating to the blood, a subject that easily becomes a most important study in the light of recent researches. Scarcely less so, however, is he on the effects of disease of the kidney on the economy, which are herein set forth with great directness. The changes in metabolism, too, are presented with a clearness that make them more easily comprehended by the student than is usually the case in such works. Whatever method contributes to the better understanding of pathology is worthy of hearty commendation, since it is an integral part of the science of internal medicine. Whoever, then, presents such a book deserves the gratitude of students and practitioners alike, for developing and putting into practical use a method of teaching so valuable.

Martin's manual is preëminently a work of this character and, unless we greatly misjudge the acumen of teachers, it will receive their commendation, and so be placed high up on the list of textbooks in the college announcements.

THE PRACTICAL MEDICINE SERIES OF YEAR BOOKS. Ten volumes. Issued under the general editorial charge of GUSTAVUS P. HEAD, M.D., Professor of Laryngology and Rhinology in the Chicago Post-Graduate Medical School. Volume II. General Surgery. Edited by John B. Murphy, M.D., Professor of Surgery, Northwestern University Medical School. Duodecimo, pp. 566. Chicago: The Year Book Publishers. 1903. (Price, $1.50; entire series, $5.50, payable in advance.)

The editor of this volume has displayed his usual surgical judgment in selecting and arranging an excellent group of subjects for presentation to the general practitioner. It would be well for every such person to read with care what is herein said in regard to anesthesia and operative technic. Murphy presents a case, with two plates, of multiple sarcoma of the entire body surface which is of rare interest; also one of carcinoma of the thyroid with secondary carcinoma of the chest wall and femur with photographic illustrations. Mitchell's operation for hemorrhoids, and May's operation for umbilical hernia, are among the operations of interest described and illustrated. The book is full of practical thoughts and suggestions which every one called upon to practise surgery will appreciate.

